# Different Signaling Pathways Define Different Interferon-Stimulated Gene Expression during Mycobacteria Infection in Macrophages

**DOI:** 10.3390/ijms20030663

**Published:** 2019-02-03

**Authors:** Xinying Zhou, Jiahui Yang, Zelin Zhang, Lijie Zhang, Bo Zhu, Linmiao Lie, Yubin Huang, Rui Ma, Chaoying Zhou, Shengfeng Hu, Qian Wen, Li Ma

**Affiliations:** Institute of Molecular Immunology, School of Laboratory Medicine and Biotechnology, Southern Medical University, Guangzhou 510515, China; zxyforever@smu.edu.cn (X.Z.); ivyyangjiahui@163.com (J.Y.); zhangzelinsmu@126.com (Z.Z.); zhanglijie20190201@163.com (L.Z.); zhuboyeah@163.com (B.Z.); llmfree@163.com (L.L.); yh280@student.le.ac.uk (Y.H.); ma8059298@gmail.com (R.M.); zhouchao@smu.edu.cn (C.Z.); hushengfeng@smu.edu.cn (S.H.); wencaoxi@smu.edu.cn (Q.W.)

**Keywords:** *Mycobacterium tuberculosis* (Mtb), interferon-stimulated genes (ISGs), macrophages (Mϕs), signaling pathways

## Abstract

Tuberculosis (TB) caused by *Mycobacterium tuberculosis* (Mtb) represents one of the greatest threats to human health., Interferons (IFNs) in combination with the first-line of anti-TB drugs have been used for treating TB for decades in the clinic, but how Mtb infection regulates interferon-stimulated genes (ISGs) in human macrophages (Mϕs) remains unknown. In this study, we investigated the expression-signature and associated innate signaling mechanisms of ISGs in Mtb-infected human monocyte-derived Mϕs (hMDMs) and THP-1-derived Mϕs (THP-1-Mϕs). Among 28 of the detected ISGs, 90% of them exerted a significant increase in Mtb-infected Mϕs. Additionally, we found that cytosolic cyclic (GMP-AMP) synthase (cGAS), toll-like receptor-2 (TLR-2) and TLR-4 signaling pathways participated in ISG induction. Their downstream elements of TANK-binding kinase 1 (TBK1), nuclear factor-kappa B (NF-κB), mitogen-activated protein kinase (MAPK), and Janus kinase-signal transducer and activator of transcription (JAK-STAT) were selectively involved in Mtb-mediated ISG production. Finally, the numerous types of ISG expression in hMDMs of TB patients were more susceptible to restimulation of Mtb infection or/and IFN treatment than that of healthy people. Hence, different signaling pathways define different ISG expression during Mtb infection and this helps to illustrate how ISGs are elucidated and to better understand the host immune responses to Mtb infection in Mϕs.

## 1. Introduction

*Mycobacterium tuberculosis* (Mtb) infection, the main cause of tuberculosis (TB), represents one of the most abundant infectious challenges to human health. The mortality of TB is high mainly due to poor sanitation, immunosuppression because of malnutrition, co-infection with the human immunodeficiency virus (HIV), and in particular burgeoning drug resistance [1]. Interferon (IFN) treatment is used as an attractive approach to protect against TB by regulating multi-tiered innate antimicrobial mechanisms and adaptive immunity, since it is less prone to develop drug resistance than direct antimycobacterial drugs [2,3]. Different types of host and various immune responses of IFNs make IFN signaling exert contradictive effects on Mtb infection, despite IFNs having been used for treating TB [4,5,6,7].

Although Mtb infection spreads to almost one-third of the population globally, only 10% of latent infections develop active TB, mainly due to the effective innate and subsequent adaptive host immune responses [8]. Among these host defense mechanisms, macrophages (Mϕs) represent the first line of anti-mycobacterial immune system, although they serve as the predominant habitat for Mtb infection and proliferation at the same time [9]. Upon Mtb infection, in addition to phagocytic activity and ability to present antigens to T-cells, Mϕs rapidly react by developing antimycobacterial immune response highly dependent on the production of cytokines, in particular IFNs [10]. Once secreted from infected cells, IFNs, induced in an autocrine and paracrine fashion, bind to their specific receptors and activate the Janus kinase-signal transducer and activator of transcription (JAK-STAT) factors of a subset of IFN-stimulated genes (ISGs), which play an important role in combating various pathogen infections and regulating host immune responses [4,11]. We assume that the different response of ISGs may be the key determinant for TB progression and an illustration of the paradoxical effects generated by IFN signaling in Mtb infection. Therefore, in Mϕs we investigated the effect and mechanism-of-action of Mtb infection on 28 ISG production responding to virus infection [11].

Triggering of the function in concert to recognize, respond, and activate the “appropriate” immune responses by Mtb infection is thought to occur as a consequence of ligation of distinct pattern recognition receptors (PRRs) [8]. In Mϕs, cytosolic cyclic GMP-AMP synthase (cGAS) DNA sensing pathway and endosomal toll-like receptor 2 (TLR-2), TLR-4 and TLR-9 have been reported as playing key roles in host defense on Mtb infection [12,13]. Subsequently, Mtb can target their downstream cellular pathways including TANK-binding kinase 1 (TBK1), nuclear factor kappa beta (NF-κB), mitogen-activated protein kinase (MAPK), and JAK-STAT signaling pathways, which are responsible for eliciting immune responses [8,14,15,16,17]. However, whether these intracellular innate signals downstream of PRR signaling pathways are involved in ISG production during Mtb infection remains unknown.

Recently, Mϕs have been recognized as innate immune cells with trained immunity, which can elicit innate immune memory by infectious stimuli, in particular *Mycobacterium bovis* (BCG) vaccine [18]. Several monocytes-secreted cytokines including Interleukin (IL)-1β, IL-6, and TNF-α and even chemokines including C-X-C motif chemokine (CXCL)9, CXCL10, and CXCL11 exerting potent immune function have been found with association of trained immunity in mycobacterial growth inhibition [19]. As ISGs are critical innate immune effectors of IFN signaling and IFN-γ primarily promotes antimicrobial effects in Mϕs [4], we investigated whether ISG production in Mtb infection or/and IFN-γ treatment could be induced after secondary stimulation (restimulation) in Mϕs of TB patients compared to healthy people.

In this study, we investigated gene expression profiles of 28 ISGs in human monocyte-derived Mϕs (hMDMs) and THP-1-derived Mϕs (THP-1-Mϕs) during Mtb infection. We further demonstrated that different Mtb-induced ISGs were dependent on the key element downstream of cyclic (GMP-AMP) synthase-stimulator of interferon genes (cGAS-STING), TLR-2 and/or -4 signaling pathways, diversely. In addition, we characterized the susceptibility of ISG induction to Mtb infection and/or IFN-γ treatment in hMDMs of TB patients and healthy people. This study investigates the effect and mechanism of ISG production in response to Mtb infection and helps to illustrate how ISGs are elucidated and to better understand the host immune responses to Mtb infection in Mϕs.

## 2. Results

### 2.1. Mtb Infection Facilitates a Subset of ISG Production in Mϕs

**Figure 1:**
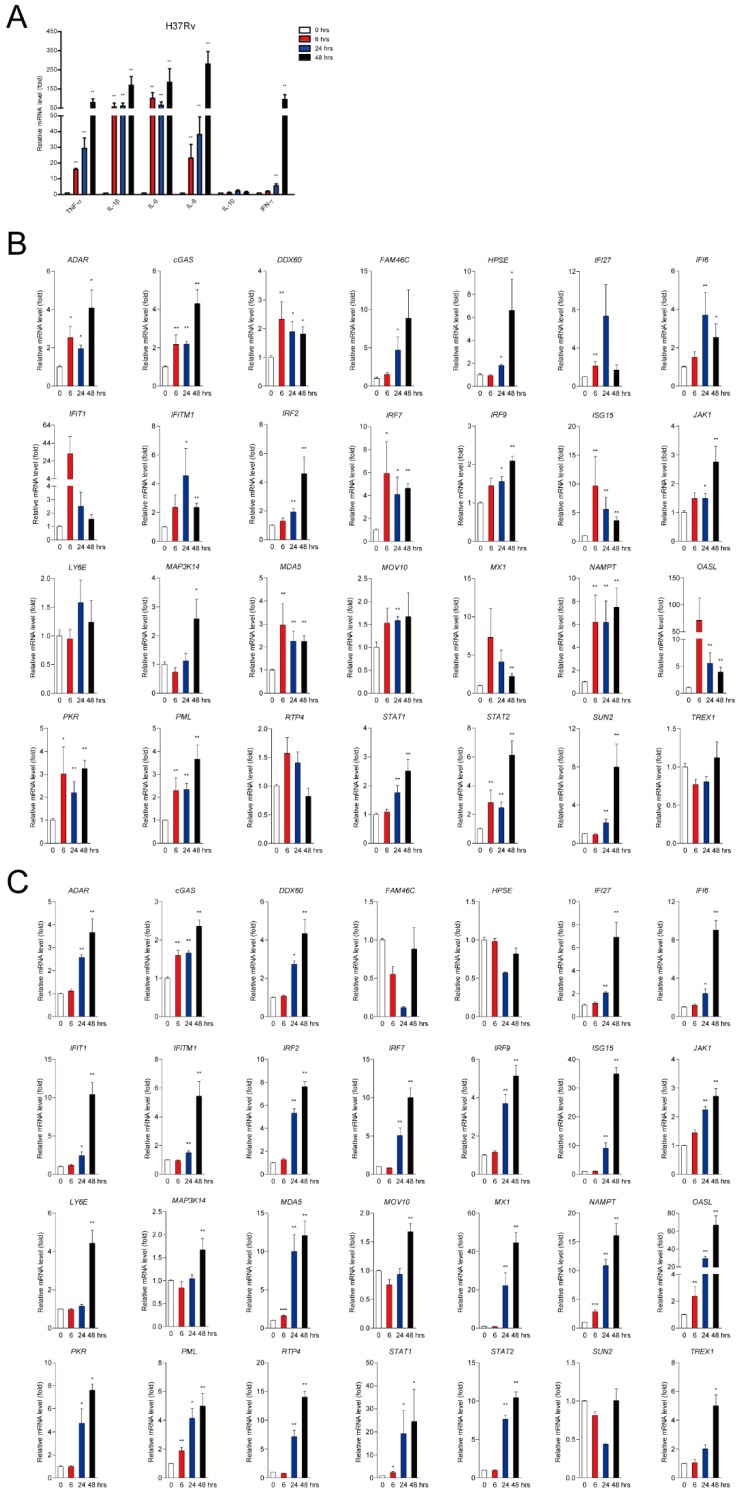
Mtb infection enhances cellular cytokines production and a subset of interferon stimulated genes (ISGs) production in Mϕs. (**A**) TNF-α, IL-1β, IL-6, IL-8, IL-10. and IFN-γ were detected by quantitative real-time-polymerase chain reaction (qRT-PCR) with H37Rv infection (multiplicity of infection (MOI) = 2) for 0 (white bars), 6 (red bars), 24 (blue bars) and 48 (black bars) h in human monocyte-derived Mϕs (hMDMs). (**B,C**) Transcript levels of 28 ISGs in hMDM (**B**) and THP-1-Mϕs (**C**) upon virulent Mtb strains H37Rv infection (MOI = 2). Data are expressed as mRNA fold change relative to uninfected cells. glyceraldehyde 3-phosphate dehydrogenase (GAPDH) served as an internal reference. (Data presented as mean ± SEM, *n* = 3 independent experiments each with 2 replicates). Infection time was indicated as 0 (white bars), 6 (red bars), 24 (blue bars) and 48 (black bars) h. * *p* < 0.05 and ** *p* < 0.01 were considered as GAPDH statistically significant.

Mϕs are the major habitat for Mtb infection and serve as the first line of innate immunity. We applied hMDMs and human cell line derived THP-1-Mϕs to evaluate the effect of Mtb infection on ISG expression. As expected, several cytokines including TNF-α, IL-1β, IL-6, IL-8, and IFN-γ were significantly increased in a time-dependent manner upon virulent Mtb strain H37Rv infection detected by qRT-PCR in hMDMs (Figure 1A). We further detected 28 of the ISG expression levels upon H37Rv infection at multiplicities of infection 2 (MOI = 2) for 6, 24, and 48 h and approximately 90% (25 out of 28) of the ISGs (except for LY6E, RTP4, and TREX1) were significantly increased in hMDMs (Figure 1B). Consistently, up to 90% (25 out of 28) of the ISGs (except for family with sequence similarity 46, member C (FAM46C), heparanase (HPSE), and SUN2) exerted significant facilitation in THP-1-Mϕs within 48 h infection of H37Rv (Figure 1C). Similarly, up-regulation of cytokines was also observed in hMDMs upon *Mycobacterium bovis* BCG infection (Figure S1A). Subsequently, we observed that BCG infection promoted the majority (24 out of 28) of the ISG production (except for HPSE, LY6E, MAP3K14, and TREX1) induction in hMDMs in a time-dependent manner (Figure S1B). These results demonstrated that mycobacterial infection significantly increased a subset of ISG production in human Mϕs.

### 2.2. ISG Production is Dependent on cGAS-STING and TLR-2/4 Signaling Pathways

**Figure 2:**
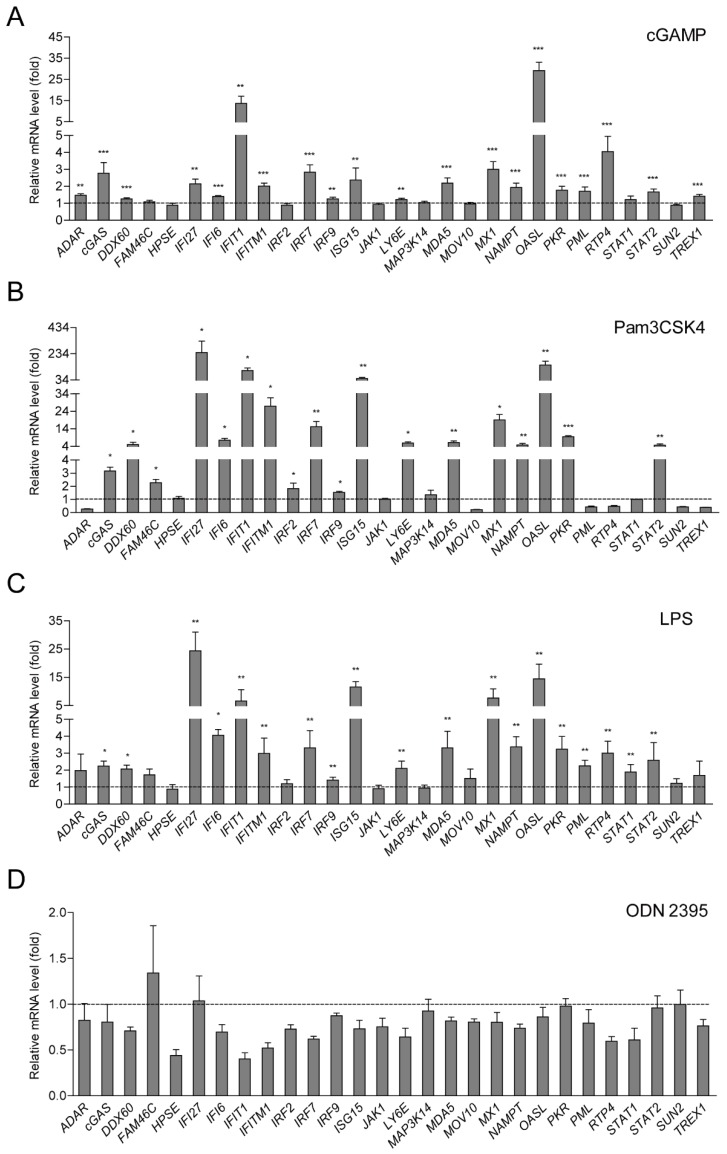
cGAS-STING and TLR-2 and -4, but not TLR-9 signaling pathways are involved in ISG induction in hMDM. (**A**) cGAMP inoculated with lipo2000 as an agonist of cGAS-STING pathway, (**B**) Pam3CSK4 as TLR-2 agonist, (**C**) lipopolysaccharide (LPS) as TLR-4 agonist and (**D**) ODN2395 as TLR9 agonist were treated with hMDMs for 24 h. Data are expressed as mRNA fold change relative to untreated cells. GAPDH served as an internal reference. (Data presented as mean ± SEM, *n* = 3 independent experiments each with 2 replicates). * *p* < 0.05, ** *p* < 0.01 and *** *p* < 0.001 were considered as statistically significant.

Mtb infection triggers a series of signaling pathways and activates a subset of innate immune response genes through pattern recognition receptors (PRRs) including cGAS-STING and Toll-like receptor (TLR)-2, -4, and -9 signaling pathways [12,14]. Whether these pathways take part in ISG production in Mϕs, remains unknown. In this study, cyclic GMP-AMP (cGAMP) as an agonist of cGAS-STING pathway, Pam3CSK4 as TLR-2 agonist, lipopolysaccharide (LPS) as TLR-4 agonist, and ODN2395 as TLR9 agonist were applied for further investigation. Upon inoculation of cGAMP with lipo2000 for 24 h, 71.5% (20 out of 28) ISGs showed significant increases in hMDMs (Figure 2A). We further observed that Pam3CSK4 and LPS significantly promoted 64.3% (18 out of 28) and 67.9% (19 out of 28) of ISG production in hMDMs separately (Figure 2B,C). However, ISG expression was not triggered with the treatment of ODN2395 (Figure 2D). These results demonstrated that cGAS-STING, TLR-2 and -4, but not TLR-9 signaling pathways were involved in ISG production in Mϕs.

### 2.3. Different ISGs Are Diversely Induced by TBK1, NF-κB, MAPK and JAK-STAT Signaling Pathways

**Figure 3:**
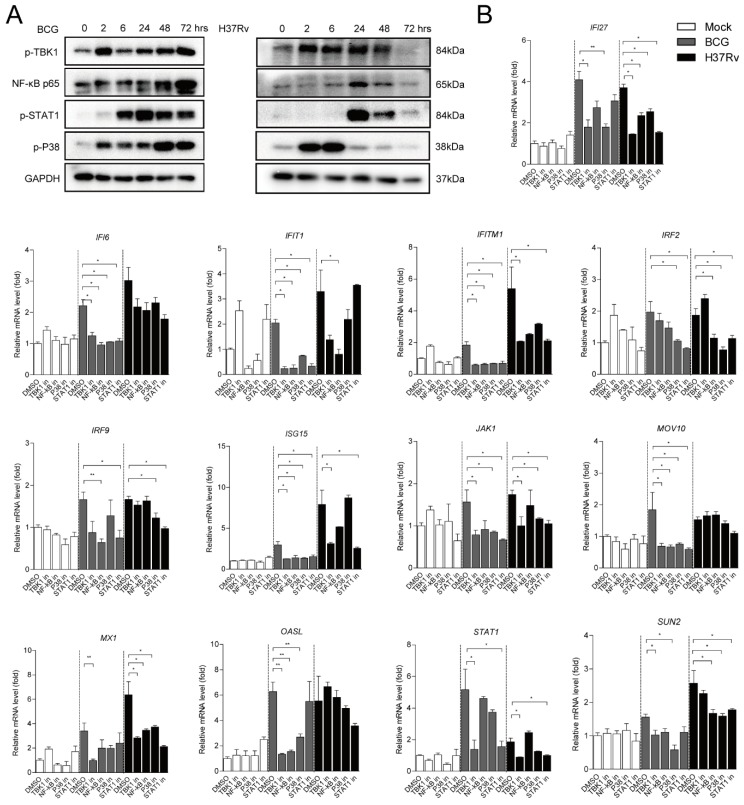
Mtb-mediated ISG production is selectively induced by TBK1, NF-κB, MAPK, and JAK-STAT signaling pathways. (**A**) Upon BCG and H37Rv (MOI = 2) infection for 0, 2, 6, 24, 48, and 72 h, p-TBK1, NF-κB p65, p-p38 MAPK, and p-STAT1 proteins were determined by Western blot analysis. GAPDH protein served as an internal reference. (**B**) Well-established inhibitors including 2 μM TBK1 inhibitor BX795, 100 μM NF-κB inhibitor JSH-23, 10 μM p38 MAPK inhibitor SB203580, and 10 μM STAT1 inhibitor fludarabine were used to treat BCG-uninfected (white columns), BCG-infected (gray columns) and H37Rv-infected (black columns) hMDMs for 24 h. Data are expressed as mRNA fold change relative to untreated cells. GAPDH served as an internal reference. (Data presented as mean ± SEM, *n* = 3 independent experiments with each 1–2 replicates). * *p* < 0.05 and ** *p* < 0.01 were considered as statistically significant.

During mycobacterial infection, cGAS-STING, TLR-2 and -4 triggered innate immune responses including TBK1, NF-κB, MAPK, and JAK-STAT-signaling pathways, which are crucial for the activation of inflammatory cytokine expression [9,15,20]. Our data showed that BCG and H37Rv infection could lead to the stimulation of key elements including p-TBK1, NF-κB p65, activated p38 kinase (p-p38 MAPK) and p-STAT1 protein levels of these signaling pathways at different time points (Figure 3A). In order to further investigate whether Mtb infection induces the 28 ISGs through these signaling pathways, several well-established inhibitors including TBK1 inhibitor BX795, NF-κB inhibitor JSH-23, p38 MAPK inhibitor SB203580, and STAT1 inhibitor fludarabine were used to treat BCG- and H37Rv-infected hMDMs for 24 h. All the inhibitors showed their biological effects to block the targeting protein levels upon Mtb infection (Figure S2A). The results demonstrated that 13 out of 24 BCG-induced ISGs and 10 out of 24 H37Rv-infected ISGs were significantly inhibited by 2 μM BX795, 100 μM JSH-23, 10 μM SB203580, or/and 10 µM fludarabine treatments in hMDMs. More specifically, BCG-induced interferon alpha-inducible protein (IFI)6, interferon-induced protein with tetratricopeptide repeats (IFIT)1, Interferon-induced transmembrane protein (IFITM)1, ISG15, and moloney leukemia virus (MOV)10 were observed attenuated by all specific inhibitors, suggesting that these signalings were involved in the 5 ISGs production induced by BCG infection. While the other 7 ISGs including IFI27, IRF2, IRF9, JAK1, 59 kDa 2’-5’-oligoadenylate synthetase-like protein (OASL), STAT1, and SUN2 were significantly and selectively blocked by certain inhibitors. In addition, H37Rv-induced 10 ISGs including IFIT1, IFITM1, ISG15, IFI27, IRF2, IRF9, JAK1, STAT1, and SUN2 were selectively inhibited by some of these inhibitors (Figure 3B). The rest of the 11 Mtb-induced ISGs showed no influence on treating with all inhibitors (Figure S2B). These findings indicated that Mtb-induced production of ISGs were selectively involved in TBK1, NF-κB, MAPK, and JAK-STAT1 signaling pathways.

### 2.4. A Number of ISGs in Mϕs of TB Patients are More Susceptible to Mycobacterial Infection or/and IFN-γ Treatment than that of Healthy People

**Figure 4:**
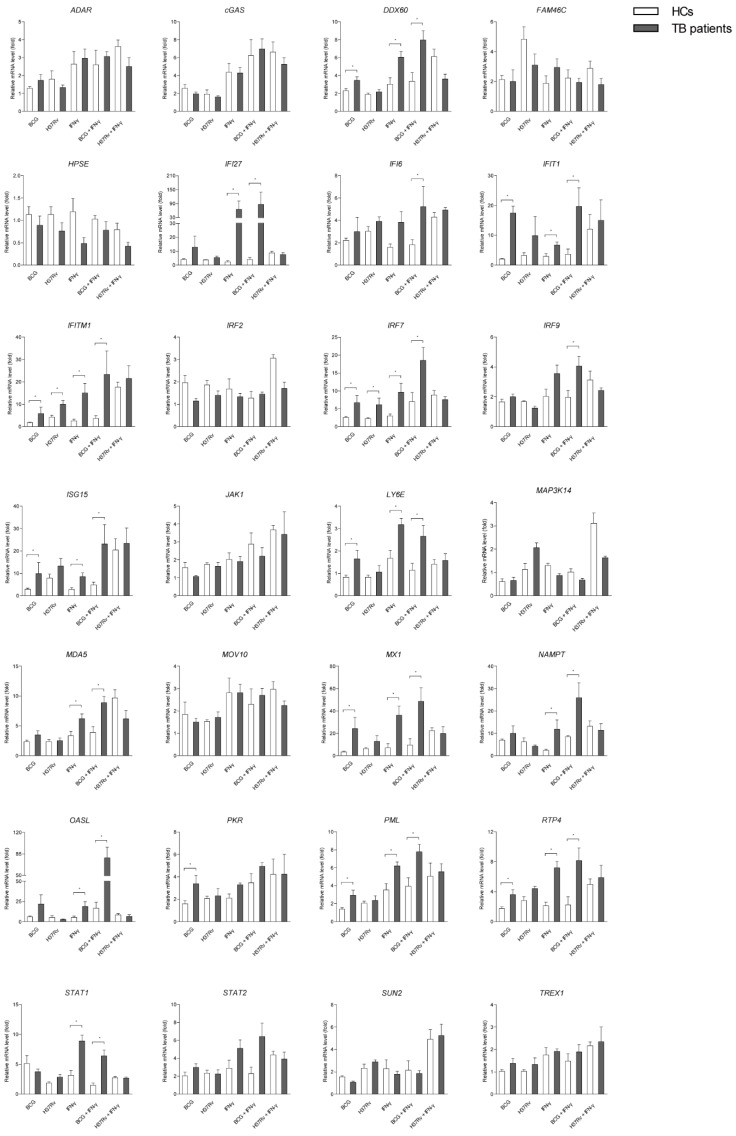
A number of ISGs in hMDMs derived from TB patients are more susceptible to mycobacterial infection and IFN-γ treatment than that from HC. 28 ISGs in hMDMs derived from TB patients (gray columns) and HC (white columns) have been detected by qRT-PCR with BCG infection, H37Rv infection (MOI = 2) or/and IFN-γ treatments (100 ng/mL) for 24 h. Data are expressed as mRNA fold change relative to untreated cells. GAPDH served as an internal reference. (Data presented as mean ± SEM, *n* = 3 independent experiments with each 1–2 replicates). * *p* < 0.05 and ** *p* < 0.01 were considered as statistically significant.

IFNs play important role in regulating innate and adaptive immune response in Mtb infection [2,4]. In our study, we demonstrated that Mtb infection and IFN-γ treatment led to a potent intracellular ISG induction in human Mϕs derived from both healthy people and TB patients. Surprisingly, 10 out of 28 ISGs (DDX60, IFIT1, IFITM1, IRF7, ISG15, LY6E, interferon-induced GTP-binding protein Mx1 (MX1), protein kinase R (PKR), promyelocytic leukemia (PML), and RTP4) induced by BCG infection and two of them (IFITM1 and IRF7) induced by H37Rv presented a further enhancement in hMDMs isolated from TB patients compared with healthy people. Consistently in TB patients, IFN-γ treatment significantly increased 14 ISGs (DDX60, IFI27, IFIT1, IFITM1, IRF7, ISG15, LY6E, melanoma Differentiation-Associated protein 5 (MDA5), MX1, Nicotinamide phosphoribosyltransferase (NAMPT), OASL, PML, RTP4, and STAT1). Upon BCG infection but not H37Rv infection with IFN-γ treatment, 15 of them (DDX60, IFI27, IFI6, IFIT1, IFITM1, IRF7, IRF9, ISG15, LY6E, MDA5, MX1, NAMPT, OASL, PML, and STAT1) exhibited a synergistic increase in TB patients (Figure 4). These results demonstrated that a subset of ISGs in hMDMs derived from TB patients is more susceptible to mycobacterial infection or/and IFN-γ treatments compared to healthy people.

## 3. Discussion

It is increasingly appreciated that IFNs not only elicit antiviral response but also manipulate the host response to Mtb infection [21]. Although, IFNs or a combination with anti-bacterial drugs were widely used to cure TB patients in the clinic, multifaceted activity of IFNs has been reported on Mtb infection [2]. For example, type I IFNs have been reported supporting Mtb infection in the majority of cases, while IFN-γ exhibits a protective function against Mtb infection [4,7]. During the infection, despite IFNs gaining the cross-talk between some key inflammatory mediators or cytokines in some circumstances, the transcription of hundreds of ISGs downstream of IFN signaling are the particular requirements for effective host defense [11,22]. Although normally ISGs act as host protective immune effectors fighting against pathogen infection, part of them can also be employed by tricky pathogens to obliterate immune response and help them survive in the host [11,23]. Therefore, the diverse and paradoxical effects of IFNs on Mtb infection can be caused by different types and various functions of ISGs. It becomes very important to define how Mtb infection affects the expression of ISGs. It has been reported that more than 380 human ISGs have the ability to inhibit the replication of several viruses. It has been found that a sub set of ISGs including HPSE, MDA5 (also known as IFIH1) and IFITMs, DDX60, IFI6, MAP3K14, MOV10, NAMPT (also known as PBEF1), OASL, RTP4, TREX1, and UNC84B (also known as SUN2) play an important role in anti-viral effects. Several ISGs, including ADAR, FAM46C, and LY6E, enhanced the replication of certain viruses. These 16 ISGs were found to be important for virus infection. In addition, a further 12 ISGs of 380 tested ISGs including STAT1, STAT2, JAK1, IRF9, IRF2, IRF7 are key elements of IFN signaling, and classical and well-known ISGs such as ISG15, PKR, MX1, IFIT1, PML, IFI27 play key roles in viral or bacterial infections [11]. Thus, we investigated whether these 28 ISGs would response to Mtb infection.

Recent studies have identified blood based profiling of IFN inducible genes as the most striking characteristic signature in TB patients [20]. Wang et al. identified a transcriptional signature involving 86 genes in blood neutrophils from patients infected with Mtb, which consisted of transcripts induced by type I and type II IFNs; the observed expression pattern provides a potential diagnostic tool for TB patients [24]. In human dendritic cells (DCs), selective expression of type I IFN genes have been detected in association with Mtb infection [25]. A few ISGs have been identified up-regulated in PBMCs, but without systematical analysis of a subset of ISGs [5]. Another study of a unique gene expression profile of 51 genes demonstrated that some up-regulated genes might account for the inflammatory condition in the alveolar Mϕs (AMs) of TB patients, but others were down-regulated, suggesting an altered control of Mtb infection [26]. Mϕs play decisive roles in host responses to intracellular Mtb infection and represent the forefront of innate immune defense against bacterial invaders [27]. In the present study, we analyzed the expression of 28 ISGs and demonstrated that approximately 90% of them were significantly increased by BCG and H37Rv in human Mϕs. The difference in the colony-forming unit (CFU) between BCG and H37Rv at MOI = 2 in both hMDM and THP-1-Mϕs after 0, 6, 24, and 48 h post infection is inconspicuous (data was not shown), indicating no difference in percentage of infected Mϕs between BCG and H37Rv infection. Thus we speculate that the different levels of ISGs induction produced by BCG and H37Rv might be due to their different level of virulence.

Once encountered, Mϕs recognize Mtb by vesicular or cytoplasmic PRRs in particular cGAS-STING system and TLR-2, -4 and -9, which are critical to Mtb infection [16,28,29]. For example, cGAS-STING has been reported to contribute to type I IFN response to Mtb infection [28]. Several lines of evidence have suggested that Mtb infection can recognize TLR-2 in producing pro-inflammatory signals, and thus TLR-2 serves as a protective factor against infection [16]. Studies using a mice model demonstrated that TLR-4 plays a protective role in host defense and exerts an increased susceptibility to Mtb infection in vivo [16]. In addition, TLR-9 has been recognized as a protective function in host defence to Mtb infection, and may cooperate with TLR-2 signaling [16]. Thus, multiple TLRs can play roles in protection against mycobacterial infection, particularly in the acute phase of infection. In this study, we showed that ISGs were induced upon direct infection of BCG and H37Rv in vitro in hMDMs. However, whether Mtb infection mediated ISG induction is associated with PRRs of cGAS-STING, TLR-2, -4 and -9 signaling pathways, remains unknown. We treated hMDMs with cGAS-STING agonist cGAMP, TLR2 agonist Pam3CSK4, TLR4 agonist LPS, and TLR9 agonist ODN2395. As the results show, 71.5%, 64.3%, and 67.9% of detected ISGs were significantly facilitated with treatment of cGAMP Pam3CSK4 and LPS respectively, but none of them increased on treating with ODN2395. These data demonstrated that Mtb infection induced ISGs were largely dependent on cGAS-STING, TLR-2 and -4, but not TLR-9 signaling pathways.

Subsequently these pathways modulate the induction of hundreds of host genes through a complex network of signaling that enables the appropriate response to Mtb infection [13]. Among this signaling, TBK1, NF-κB, MAPK, and JAK-STAT are strongly associated with activation of the host defense against Mtb infection, including various pro-inflammatory responses executed by expression and/or secretion of cytokine release of anti-microbial effectors [30,31,32]. Researchers have demonstrated that TBK-1 is required for IL-1β-induced autophagic elimination of Mtb infection in Mϕs [30]. Another study indicated that Mtb is able to induce the production of TNF-α and IL-1β in RAW264.7 cells and that it activates the NF-κB pathway through TLR2-mediated signaling, thereby contributing to the induction of IRF-1, one of the most potent ISGs against viral infection [31]. Additionally, one study demonstrated that Mtb can activate RAW264.7 and trigger IL-6 and IL-12 p40 production via ERK, p38 MAPK, and NF-κB signaling pathways [32]. Furthermore, JAK-STAT signaling which elicits several innate immune responses can be responsible for controlling Mtb infection [6]. Here we exploited TBK1 inhibitor BX795, NF-κB inhibitor JSH-23, p38 MAPK inhibitor SB203580, and STAT1 inhibitor fludarabine to block their biological targeting proteins in hMDMs and detected their effects on Mtb-induced ISG expression. Our data demonstrated that Mtb infection induced these ISGs selectively through TBK1, NF-κB, MAPK, and JAK-STAT signaling pathways. These findings enhance our understanding of the Mtb-mediated innate immune response of IFN signaling in Mϕs, and provide a clue that these ISGs may play vital roles in host defense against invading Mtb, although it requires further investigation.

Recently, several studies focused on innate immune memory termed trained innate immunity induced by infectious stimuli, including bacterial or fungal cells and their components as well as viruses or even parasites in monocytes, Mϕs, and natural killer (NK) cells [18]. In Mtb infection, trained innate immunity of monocytes induced the response of the cells after secondary stimulation (restimulation) with a number of cytokines and was responsible for bacterial control [19]. In this study, numerous ISGs in hMDMs of TB patients were more susceptible to Mtb infection or/and IFN-γ treatment than that of healthy people. These results demonstrated that Mϕs are able “to acquire” enhanced capability to respond to Mtb stimuli and IFNs treatment and could also amount to some kind of memory. These results might provide some evidence that in TB patients it is much easier to stimulate effective immune response to secondary Mtb infection than in healthy people.

Together, we demonstrated that most of the detected ISGs were up-regulated upon Mtb infection in vitro in human Mϕs. This Mtb-mediated ISG production might be associated with cGAS-STING, TLR-2 and -4, but not TLR-9 signaling pathways. In addition, multiple cellular pathways including TBK1, NF-κB, MAPK, and JAK-STAT were selectively involved in eliciting ISG transcription, although innate immunity has been considered to be more primitive and less sophisticated compared to the adaptive one. We demonstrated that numerous ISGs in hMDMs derived from the PBMCs of TB patients were more susceptible to mycobacterial infection or/and IFN-γ treatment than that of healthy people. Hence, different signaling pathways define different ISG expression during Mtb infection and this may help to illustrate how ISGs are elucidated and to better understand the host immune responses to Mtb infection in Mϕs.

## 4. Materials and Methods

### 4.1. Study of Populations/Participants

Four patients with active pulmonary tuberculosis and six healthy controls (HC) were recruited for this study. The healthy people did not have any past history or present symptoms of TB. Patients with active pulmonary TB were recruited from the Guangzhou Chest Hospital, Guangzhou, China. HC subjects were chosen from consenting adults drawn from the same background and locality as the TB patients.

The study was carried out in accordance with the recommendations of the International Committee of Medical Journal Editors and the ethics committee of the Southern Medical University with written informed consent from all subjects (project identification code is 81801584 from National Natural Science Foundation of China, 1 January 2019). All subjects gave written informed consent in accordance with the Declaration of Helsinki. The protocol was approved by the ethics committee of the Southern Medical University.

### 4.2. Cell Culture of hMDMs and THP-1-Mϕs and Mtb Infection

An amount of 5 mL of venous blood was collected and mixed with 1 volume of phosphate buffer solution (PBS), layered onto a Lymphoprep™ (Alere Technologies AS, Oslo, Norway) and isolated by isopycnic gradient centrifugation for 20 min, 25 °C. peripheral blood mononuclear cells (PBMCs) were collected and removed to a new tube and washed with phosphate-buffered saline (PBS) three times, each time centrifuged at 800 *g* for 10 min at 25 °C to discard the supernatant. Harvested PBMCs were resuspended with 5 mL PBS and cell numbers were adjusted to 1 × 10^6^/mL. hMDM derived from PBMCs isolated from peripheral blood of healthy volunteers and TB patients and were placed on 6-well culture plates in the Roswell Park Memorial Institute (RPMI) in 1640 medium (Corning, New York, NY, USA) containing 10% FBS (Corning, USA). After culturing in a 37 °C, 5% CO_2_ incubator, the suspension cells were discarded after 24 h, and were induced for 7 days by adding 100 ng/mL human granulocyte macrophage colony stimulating factor (GM-CSF) (Pepro Tech, New Jersey, NJ, USA). The medium was changed every two days. THP-1 cells (CELLCOOK, Guangzhou, China) were cultured in RPMI 1640 medium containing 10% FBS by adding 100 ng/mL phorbol-12-myristate-13-acetate (PMA) (Pepro Tech, New Jersey, NJ, USA) for 48 h incubation. The adherent cells were THP-1-Mϕs.

H37Rv (American Type Culture Collection) and BCG (SINOPHARM, Beijing, BJ, China) were cultured in Middlebrook 7H9 broth with 10% BBL Middlebrook oleic acid–albumin–dextrose–catalase (OADC) at 37 °C under 5% CO_2_. Mycobacteria were ground to generate a single bacterium suspension in RPMI 1640. Then both PBMCs and THP-1-Mϕ cells were placed on 12-well cell culture plates (CELLTER, China) and were continuously infected with H37Rv and BCG at MOI of 2 at the corresponding time points.

### 4.3. Reagents

hMDMs were inoculated with cGAS-STING agonist cGAMP (Invivogen, Life Technologies, Carlsbad, CA, USA) of 20 μg/mL, TLR2 agonist Pam3CSK4 (Invivogen, Life Technologies, Carlsbad, CA, USA) of 66.2 nM, TLR4 agonist lipopolysaccharide (LPS) (Sigma, USA) of 100 ng/mL, and TLR9 agonist ODN 2395 (Invivogen, Life Technologies, Carlsbad, CA, USA) of 5 μM for 24 h. ODN 2395 and cGAPM transfection were carried out with 5 μL Lipofectamine 2000 (Life Technologies, Carlsbad, CA, USA) per ml medium. TBK1 inhibitor BX795 (Selleckchem, Houston, TX, USA) of 2 μM, NF-κB inhibitor JSH-23 (Selleckchem, Houston, TX, USA) of 100 uM, p38 MAPK inhibitor SB203580 (Selleckchem, Houston, TX, USA) of 10 μM and STAT1 inhibitor fludarabine (MCE, Monmouth Junction, NJ, USA) of 10 μM were applied respectively. Human IFN-γ (Pepro Tech, Rocky Hill, USA) of 100 ng/mL was used to treat hMDMs for 24 h to induce ISG production.

### 4.4. RNA Extraction and Reverse Transcription

RNA was extracted from PBMCs/hMDMs derived from TB patients and HC and THP-1-Mϕ cells, using Total RNA Kit I (200) (OMEGA, Norcross, GA, USA) according to the operating instructions. After the RNA concentration was measured using NanoDrop 2000 (Thermo, Carlsbad, CA, USA), cDNA was synthesized using TransScript One-Step gDNA Removal and cDNA Synthesis SuperMix (TransGen Biotech, Beijing, China) according to the protocol.

### 4.5. Real Time PCR Quantitation of Cytokine mRNA

The mRNA expression of ISGs and GAPDH were assessed by SYBR^®^ Premix Ex Taq™ II (Tli RNaseH Plus) (TaKaRa, Beijing, China) on Mastercycler ep realplex4 (Eppendorf, Hamburg, Germany). The PCR conditions included an initial step at 95 °C for 120 s, followed by 45 cycles of amplification and quantification (95 °C for 15 s, 60 °C for 15 s), followed by a final extension at 68 °C for 20 s. GAPDH was used as an internal control. Relative gene expression levels were calculated using the 2^−ΔΔ*C*t^ method. Full primers are shown in Table S1.

### 4.6. Protein Preparation and Western Blot Analysis

Cells were harvested in lysis buffer containing 455 mM Tris (pH 6.8) (Sangon Biotech, Shanghai, China), 41.6 mM SDS (Zhuosheng Biotech, Shanghai, China), 26.9 μM, 30% (*v*/*v*) glycerol (SIGMA, Merck KGaA, Darmstadt, Germany), and 10 μM DL-Dithiothreitol (DTT) (SIGMA, Merck KGaA, Darmstadt, Germany). Proteins were separated by SDS–PAGE and transferred to polyvinylidene (PVDF) membranes (Merck KGaA, Darmstadt, Germany) by the wet transfer method. Membranes were blocked with 5% (*w*/*v*) BSA (SIGMA, USA) in PBS containing 0.1% (*v*/*v*) Tween-20 (PBS-T) for 1 h before incubation with primary antibodies at a dilution of 1:1000 at 4 °C overnight. The antibodies used were anti-GAPDH (17AF0412, ZSGB-BIO, Beijing, China), anti-phospho (Ser172)-TBK1/NAK (D52C2, Cell Signaling Technology, Danvers, MA, USA), anti-phospho (Ser536)-NF-κB p65 (93H1) (3033, Cell Signaling Technology, USA), anti-phospho (T180/Y182)-p38 MAPK (D39E) (4511, Cell Signaling Technology, USA) and anti-phospho (Tyr701)-STAT1 (9167, Cell Signaling Technology, USA). Followed by incubation with secondary antibodies goat anti-rabbit at a dilution of 1:1,000 or anti-mouse IgG-horseradish peroxidase conjugates at a dilution of 1:3000 (ZSGB-BIO, Beijing, China) for 1 h at room temperature. After three washes with PBS-T for 30 min the immunoblots were visualized by enhanced chemiluminescence (ECL; Thermo Fisher Scientific, Carlsbad, CA, USA) on FluorChem Systems (ProteinSimple, California, CA, USA)

### 4.7. Statistical Analysis

Results are shown as means ± SD of triplicate experiments. Statistical analyses were performed using unpaired t test and the unpaired Mann–Whitney U test. * *p* < 0.05, ** *p* < 0.01 and *** *p* < 0.001 were considered as statistically significant. Statistics were performed with Prism 5.0 (GraphPad software 7.0, lnc., Cary, NC, USA).

## Supplementary Materials

Supplementary materials can be found at http://www.mdpi.com/1422-0067/20/3/663/s1.
