# Supplementary material for: Different Signaling Pathways Define Different Interferon-Stimulated Gene Expression during Mycobacteria Infection in Macrophages
Source: Int J Mol Sci. 2019 Feb 3;20(3):663. doi: 10.3390/ijms20030663 (PMC6387094; doi:10.3390/ijms20030663)
Supplement: Supplementary file 1 [file ijms-20-00663-s001.pdf]

SUPPLEMENTARY MATERIALS

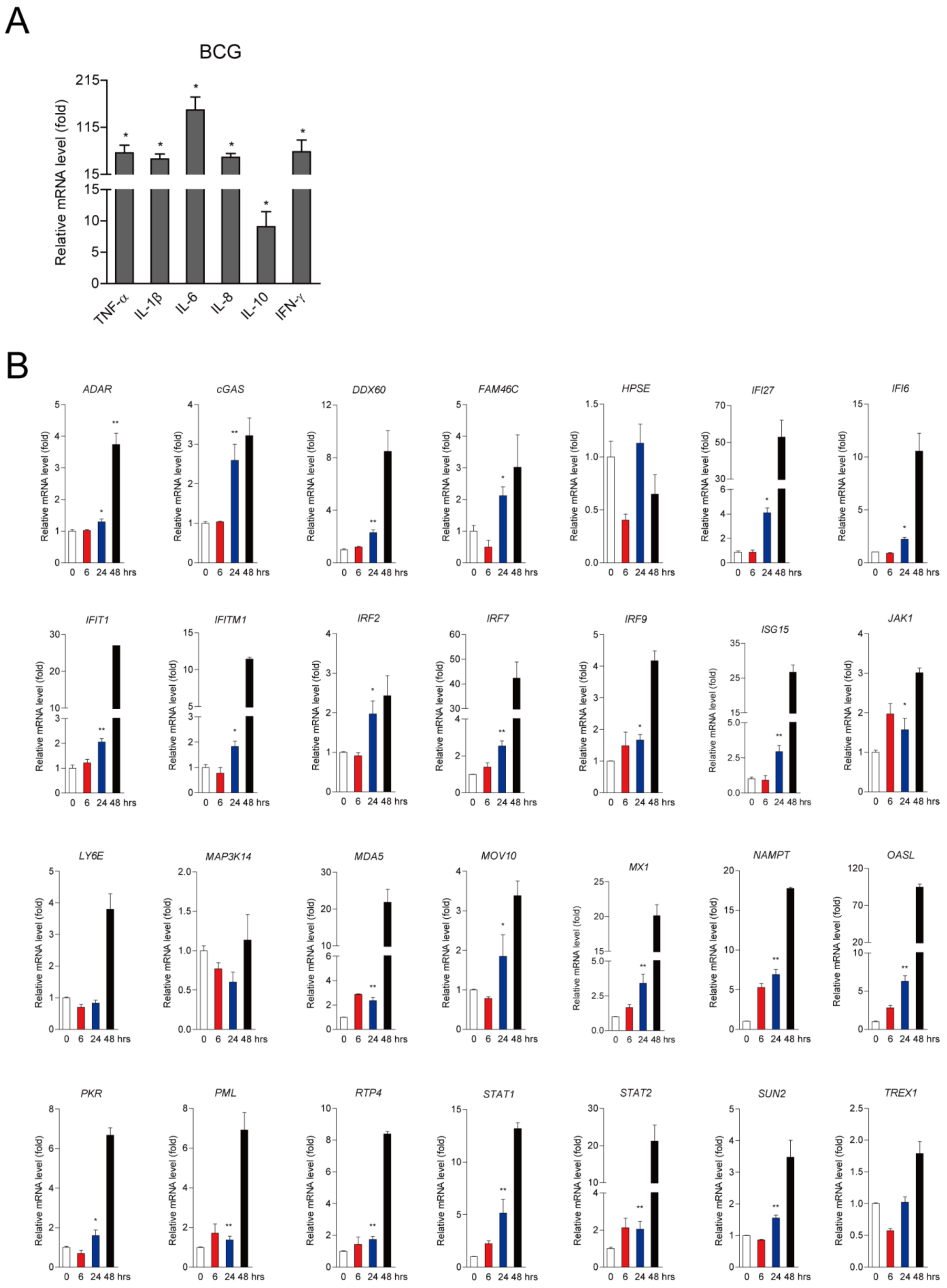

FIGURE S1. BCG infection induced cellular cytokines production and a subset of ISG production in hMDMs. **(A)** TNF-α, IL-1β, IL-6, IL-8, IL-10 and IFN-γ have been detected by qRT-PCR with BCG infection (MOI = 2) for 24 hrs in hMDMs. **(B)** Transcript level of ISGs in attenuated live culture preparation of BCG infected hMDM. Data are expressed as mRNA fold change relative to uninfected cells. GAPDH served as an internal reference. (Data presented as mean ± SEM, n = 3 independent experiments with each 2 replicates). Infection time was indicated as 0 (white bars), 6 (red bars), 24 (blue bars) and 48 (black bars) hrs. * $p < 0.05$ and ** $p < 0.01$ were considered as statistically significant.

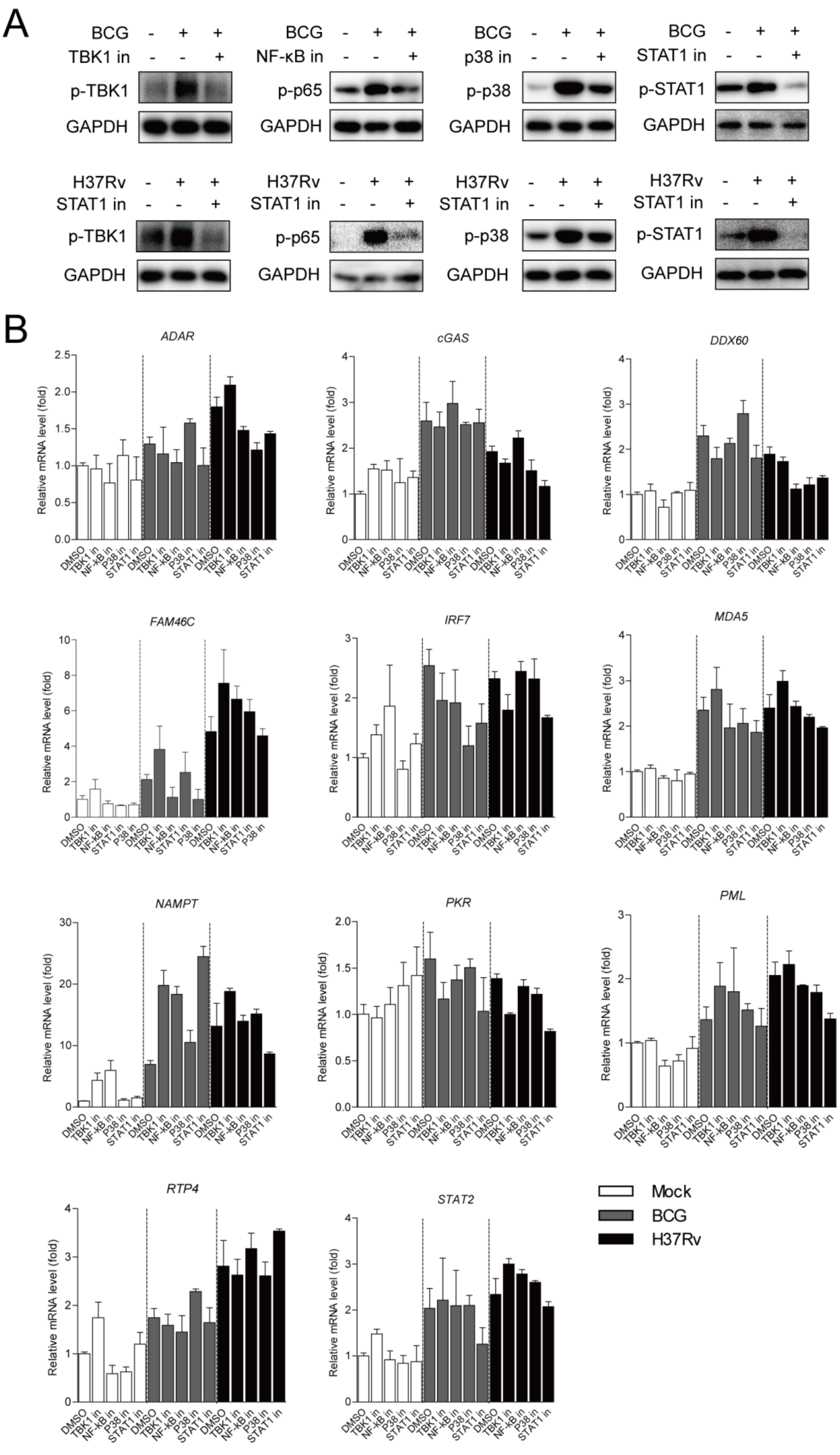
A
BCG - + +
TBK1 in - - +
p-TBK1
GAPDH
NF-κB in - - +
p-p65
p38 in - - +
p-p38
STAT1 in - - +
p-STAT1
H37Rv - + +
STAT1 in - - +
B
ADAR
cGAS
DDX60
FAM46C
IRF7
MDA5
NAMPT
PKR
PML
RTP4
STAT2
Relative mRNA level (fold)
DMSO
TBK1 in
NF-κB in
P38 in
STAT1 in
Mock
BCG
H37Rv

FIGURE S2. Several Mtb-mediated ISGs were not associated with TBK1, NF-κB, MAPK and JAK-STAT signaling pathways. (A) Well-established inhibitors including 2 μM TBK1 inhibitor BX795, 100 μM NF-κB inhibitor JSH-23, 10 μM p38 MAPK inhibitor SB203580, and 10 μM STAT1 inhibitor fludarabine have been used to treat uninfected, BCG-infected and H37Rv-infected hMDMs for 24 hrs. p-TBK1, p-p65, p-p38 and p-STAT1 have been detected by Western blot assay. GAPDH served as an internal reference. (B) ISG expression levels have been detected by qRT-PCR. Data are expressed as mRNA fold change relative to untreated cells. GAPDH served as an internal reference. (Data presented as mean ± SEM, n = 3 independent experiments with each 1-2 replicates).

SUPPLEMENTARY TABLE 1. List of genes whose mRNA expression was quantitated in qRT-PCR assay.

No.	Gene symbol	Full name	Primers (5' - 3')
1	GAPDH	Glyceraldehyde-3-phosphate dehydrogenase	F: GTCTCCTCTGACTTCAACAGCG<br>R: ACCACCCTGTTGCTGTAGCCAA
2	TNF	Tumor necrosis factor	F: CTCTTCTGCCTGCTGCACTTTG<br>R: ATGGGCTACAGGCTTGTCACTC
3	IL-1ß	Interleukin 1 beta	F: CCACAGACCTTCCAGGAGAATG<br>R: GTGCAGTTCAGTGATCGTACAGG
4	IL-6	Interleukin 6	F: AGACAGCCACTCACCTCTTCAG<br>R: TTCTGCCAGTGCCTCTTTGCTG
5	IL-8	Interleukin 8	F: GAGAGTGATTGAGAGTGGACCAC<br>R: CACAACCCTCTGCACCCAGTTT
6	IL-10	Interleukin 10	F: TCTCCGAGATGCCTTCAGCAGA<br>R: TCAGACAAGGCTTGGCAACCCA
7	ADAR	Adenosine deaminase, RNA specific	F: TCCGTCTCCTGTCCAAAGAAGG<br>R: TTCTTGCTGGGAGCACTCACAC
8	cGAS	Cyclic GMP-AMP synthase	F: AGGAAGCAACTACGACTAAAGCC<br>R: CGATGTGAGAGAAGGATAGCCG
9	DDX60	Dexd/H-box helicase 60	F: GGTGTTTTCACCAGGGAGTATCG<br>R: CCAGTTTTGGCGATGAGGAGCA
10	FAM46C	Family with sequence similarity 46 member C	F: CCTTGAACAGCAGAGGAAGTTGG<br>R: GGAGATGAGGTTCAGAGTCTGC

11	HPSE	Heparanase	F: GAATGGACGGACTGCTACCAAG<br>R: CTCCTAACCAGACCTTCTTGCC
12	IFI27	Interferon alpha inducible protein 27	F: CGTCCTCCATAGCAGCCAAGAT<br>R: ACCCAATGGAGCCCAGGATGAA
13	IFI6	Interferon alpha inducible protein 6	F: TGATGAGCTGGTCTGCGATCCT<br>R: GTAGCCCATCAGGGCACCAATA
14	IFIT1	Interferon induced protein with tetratricopeptide repeats 1	F: GCCTTGCTGAAGTGTGGAGGAA<br>R: ATCCAGGCGATAGGCAGAGATC
15	IFITM1	Interferon induced transmembrane protein 1	F: GGCTTCATAGCATTCGCCTACTC<br>R: AGATGTTCAGGCACTTGGCGGT
16	IRF2	Interferon regulatory factor 2	F: TAGAGGTGACCACTGAGAGCGA<br>R: CTCTTCATCGCTGGGCACACTA
17	IRF7	Interferon regulatory factor 7	F: CCACGCTATACCATCTACCTGG<br>R: GCTGCTATCCAGGGAAGACACA
18	IRF9	Interferon regulatory factor 9	F: CCACCGAAGTTCCAGGTAACAC<br>R: AGTCTGCTCCAGCAAGTATCGG
19	ISG15	ISG15 ubiquitin-like modifier	F: CTCTGAGCATCCTGGTGAGGAA<br>R: AAGGTCAGCCAGAACAGGTCGT
20	JAK1	Janus kinase 1	F: GAGACAGGTCTCCCACAAACAC<br>R: GTGGTAAGGACATCGCTTTTCCG
21	LY6E	Lymphocyte antigen 6 family member E	F: GACCAGGACAACTACTGCGTGA<br>R: AAGCCACACCAACATTGACGCC

22	MAP3K14	Mitogen-activated protein kinase kinase kinase 14	F: GGAATACCTCCACTCACGAAGG<br>R: CTGTGAGCAAGGACTTTCCCAG
23	MDA5	Melanoma differentiation associated gene 5	F: GCTGAAGTAGGAGTCAAAGCCC<br>R: CCACTGTGGTAGCGATAAGCAG
24	MOV10	Mov10 RISC complex RNA helicase	F: GGCAAGACTGTCACGTTAGTGG<br>R: GGAGCCTTTGACAGAGTAGGTC
25	MX1	MX dynamin like gtpase 1	F: GGCTGTTTACCAGACTCCGACA<br>R: CACAAAGCCTGGCAGCTCTCTA
26	NAMPT	Nicotinamide phosphoribosyltransferase	F: AGGGTTACAAGTTGCTGCCACC<br>R: CTCCACCAGAACCGAAGGCAAT
27	OASL	2'-5'-oligoadenylate synthetase like	F: GTGCCTGAAACAGGACTGTTGC<br>R: CCTCTGCTCCACTGTCAAGTGG
28	PKR	Dsrna-activated protein kinase R	F: CGGAAGGACACGGCATCAAGAT<br>R: GAGCCAGGAAAACCTTCTCTGC
29	PML	Promyelocytic leukemia	F: CCGTCATAGGAAGTGAGGTCTTC<br>R: GTTTTCGGCATCTGAGTCTTCCG
30	RTP4	Receptor transporter protein 4	F: GACGCTGAAGTTGGATGGCAAC<br>R: GTGGCACAGAATCTGCACTTGG
31	STAT1	Signal transducer and activator of transcription 1	F: ATGGCAGTCTGGCGGCTGAATT<br>R: CCAAACCAGGCTGGCACAATTG
32	STAT2	Signal transducer and activator of transcription 2	F: CAGGTCACAGAGTTGCTACAGC<br>R: CGGTGAACTTGCTGCCAGTCTT

33	SUN2	Sad1 and UNC84 domain containing 2	F: GGTGTGATTGGAGTGACAGAGG
			R: CTCGTAGGTCTCAGAACATCGG
34	TREX1	Three prime repair exonuclease 1	F: GCATCTGTCAGTGGAGACCACA
			R: CAGTGGTTGTGACAGCAGATGG

F: forward primer; R: reverse primer.
